# Acupuncture in adults with Chemotherapy-Induced Peripheral Neuropathy: a systematic review

**DOI:** 10.1590/1518-8345.2959.3126

**Published:** 2019-03-18

**Authors:** Amanda Fonseca Baviera, Karin Olson, Juliana Maria de Paula, Bruna Francielle Toneti, Namie Okino Sawada

**Affiliations:** 1 Universidade de São Paulo, Escola de Enfermagem de Ribeirão Preto, PAHO/WHO Collaborating Centre for Nursing Research Development, Ribeirão Preto, SP, Brazil.; 2 University of Alberta, Faculty of Nursing, Edmonton, AB, Canada.

**Keywords:** Neoplasms, Antineoplastic Agents, Acupuncture, Acupuncture Therapy, Acupuncture Points, Peripheral Nervous System Diseases., Neoplasmas, Agentes Antineoplásicos, Acupuntura, Terapia com Acupuntura, Pontos de Acupuntura, Doenças do Sistema Nervoso Periférico, Neoplasmas, Agentes Antineoplásicos, Acupuntura, Terapia de Acupuntura, Puntos de Acupuntura, Enfermedades del Sistema Nervioso Periférico

## Abstract

**Objective:**

to analyze and synthesize knowledge about the effect of acupuncture on chemotherapy-induced peripheral neuropathy symptoms in adults with cancer.

**Method:**

the method used was a Systematic Review. Potential articles were identified by searching in the PubMed of National Library of Medicine, Cumulative Index to Nursing and Allied Health Literature, Embase, Cochrane Central and Scopus. Following the Preferred Reporting Items for Systematic Reviews and Meta-Analyses strategy, 607 articles were identified. After removing the duplicates, all titles and abstracts were reviewed, and seven articles were selected for full review. After the full review, five studies were selected for inclusion.

**Results:**

of the five articles included, four were cohort studies and one was a quasi-experimental study. All articles showed that acupuncture was associated with an improvement in the peripheral neuropathy, but the type of protocol, use of medications, time of treatment, and different outcome measures made it difficult to compare the studies.

**Conclusion:**

the use of acupuncture appears to be associated with an improvement in the symptoms of chemotherapy-induced peripheral neuropathy and has no side effects. In order to improve the evidence about benefits associated with acupuncture, more experimental studies using both subjective and objective measures are needed.

## RESUMO

**Objetivo:**

analisar e sintetizar o conhecimento sobre o efeito da acupuntura nos sintomas da neuropatia periférica induzida por quimioterapia em adultos com câncer.

**Método:**

o método utilizado foi uma Revisão Sistemática. Artigos potenciais foram identificados através de pesquisa na PubMed *of National Library of Medicine, Cumulative Index to Nursing and Allied Health Literature*, Embase, Cochrane Central e Scopus. Seguindo a estratégia *Preferred Reporting Items for Systematic Reviews and Meta-Analysis* foram identificados 607 artigos. Depois de eliminar artigos duplicados, todos os títulos e resumos foram revisados, deixando sete artigos para revisão na íntegra, e após tal revisão, cinco artigos foram selecionados para inclusão no presente estudo.

**Resultados:**

dos cinco artigos incluídos, quatro eram estudos de coorte e um era um estudo quase experimental. Todos os artigos mostraram que a acupuntura estava associada a uma melhora na neuropatia periférica, mas o tipo de protocolo, o uso de medicamentos, o tempo de tratamento e as diferenças entre as medidas de desfecho dificultaram a comparação entre os estudos.

**Conclusão:**

o uso da acupuntura parece estar associado à melhora dos sintomas da neuropatia periférica induzida por quimioterapia e não tem efeitos colaterais. São necessários mais estudos experimentais usando tanto medidas subjetivas como objetivas para melhorar as evidências sobre os benefícios associados à acupuntura.

## RESUMEN

**Objetivo:**

analizar y sintetizar el conocimiento sobre el efecto de la acupuntura en los síntomas de neuropatía periférica inducida por quimioterapia en los adultos con cáncer.

**Método:**

el método utilizado fue una Revisión Sistemática. Artículos potenciales se identificaron mediante la búsqueda en PubMed *of National Library of Medicine, Cumulative Index to Nursing and Allied Health Literature*, Embase, Cochrane Central y Scopus. Siguiendo los elementos de informes *Preferred Reporting Items for Systematic Reviews and Meta-Analyses*, se identificaron 607 artículos. Después de eliminar los duplicados, se revisaron todos los títulos y resúmenes, quedando siete artículos para una revisión completa y después de la revisión completa, se seleccionaron cinco estudios para su inclusión.

**Resultados:**

de los cinco artículos incluidos, cuatro fueron estudios de cohortes y uno fue un estudio cuasi experimental. Todos los artículos mostraron que la acupuntura se asoció con una mejoría en la neuropatía periférica, pero el tipo de protocolo, el uso de medicamentos, el tiempo de tratamiento y las diferencias entre las medidas de resultado dificultaron la comparación de los estudios.

**Conclusión:**

el uso de la acupuntura parece estar asociado con una mejoría en los síntomas de la neuropatía periférica inducida por quimioterapia y sin efectos secundarios. Para mejorar la evidencia sobre los beneficios asociados con la acupuntura, se necesitan más estudios experimentales que utilicen medidas subjetivas y objetivas.

## Introduction

Chemotherapy is one of the most important treatments for cancer, but it has many adverse effects that adversely impact the patient’s quality of life. One adverse effect of chemotherapy is chemotherapy-induced peripheral neuropathy (CIPN)^1^. 

CIPN is a serious problem because it leads to difficulties in adherence to chemotherapy treatment, which may have an impact on both the patient’s daily life and the long-term outcome of the treatment. The drugs that can cause CIPN include Cisplatin, Oxaliplatin, Paclitaxel, Thalidomide, and Bortezomib^1^. Patients who receive Cisplatin, for example, may experience loss of all sensory modalities, ataxia and gait imbalance, early reduction/loss of deep reflexes, paresthesia (burning sensation, tingling), numbness (loss of sensation), among others. These symptoms may continue for months after the end of treatment^1^. The intensity and degree of severity of the symptoms depends on the drug, dose, treatment time, and other co-morbid conditions, such as Diabetes, prior exposure to neurotoxic agents, and alcohol exposure^2^. 

CIPN is diagnosed by a health care provider, usually based on the patient’s self-report, but a growing number of objective measures are becoming available. Objective measures include nerve conduction studies (NCS), neurological examinations, and cytokine assessments. A few studies on pharmacological treatments for CIPN, such as vitamin E, glutathione, and lipoic acid, have been conducted, but their quality is poor and the results are not consistent, owing largely to reliance on self-reported outcomes^1^. The primary approach to the management of CIPN is dose delays and dose reductions; thus, it is very important to diagnose CIPN as early as possible, so that the chemotherapy dose can be adjusted^2^.

 There has been a growing interest in acupuncture, a common complementary therapy, as a new intervention for CIPN. Acupuncture is an ancient practice that originated within the Traditional Chinese Medicine (TCM) in which needles are inserted into the patient’s skin at various points in the body^3^. Acupuncture results in a sensation known as *De-Qi*, which is considered fundamental to its effect, but the actual receptors and nerve fibers involved are unknown^4^. The meaning of *Qi*, which is subjective and dependent on the context and coordinates in which it is experienced, is considered to be the patient’s “energy” or “the arrival of vital energy”^5^. The mechanisms of action of acupuncture are still not fully understood, but the most commonly held hypotheses are that acupuncture leads to increased blood flow in the capillaries at the needle insertion sites, releases local opioid peptides, reduces inflammation, and stimulates specific areas of the brain^6^.

Researchers have studied the impact of acupuncture on many disorders such as musculoskeletal diseases^7^, chronic lower back pain^8^, nausea in pregnancy^9^ and headache^10^. Researchers have also shown that acupuncture is effective for the treatment of many cancer symptoms caused by chemotherapy or radiotherapy, such as nausea^11^, vomiting^11^, cancer pain^12^, hot flashes^13^, and fatigue^14^. Furthermore, recent studies^15^ ^-^ ^16^ with other modalities such as laser acupuncture and auricular therapy have demonstrated their efficacy in the management of systemic arterial hypertension and chronic spinal pain. The anatomic points chosen by the person who applies the acupuncture may vary. Some individuals follow specific acupuncture protocols while others develop specific protocols for each patient.

The objective of this review was to analyze and synthesize the knowledge about the effect of acupuncture on chemotherapy-induced peripheral neuropathy symptoms in adults with cancer. The research question for this review was: Does acupuncture reduce chemotherapy-induced peripheral neuropathy symptoms in adults with cancer? 

## Method

The method used was a Systematic Review of the literature (SR). A SR is a strategy that aims to identify, evaluate and synthesize relevant studies on a given topic, gathering evidence that responds to a specific clinical problem. Systematic reviews are used to establish evidence-based clinical practice^17^. The present review was conducted using the Preferred Reporting Items for Systematic Reviews and Meta-Analyses (PRISMA) strategy^18^, with the following inclusion and exclusion criteria:

Inclusion criteria: peer-reviewed English studies of adults (18 years old or more) diagnosed with cancer, who were able to give consent without proxy, with symptoms of CIPN diagnosed by a health care provider and treated with acupuncture (without electrical, laser or auricular stimulation).
Exclusion criteria: case series, case reports, studies with interventions that included electrical or laser stimulation and auricular acupuncture, studies with animals, studies with adults diagnosed with dementia, reviews, conference abstracts, studies with acupuncture and substances other than prescribed medications, studies about neuropathic pain only, and articles without access to the full text.

The PICO^19^ statement for this review was: P: adults with chemotherapy-induced peripheral neuropathy; I: acupuncture; C: adults with chemotherapy-induced peripheral neuropathy who did not receive acupuncture treatment; and O: improvement of chemotherapy-induced peripheral neuropathy symptoms. The databases used were: PubMed of National Library of Medicine, Cumulative Index to Nursing and Allied Health Literature (CINAHL), Embase, Cochrane Central and Scopus. The search in all databases included articles published since the beginning of each index until February 2018. The terms used in the search were:

PubMed: (“Peripheral Nervous System Diseases”[Mesh] OR (“peripheral neuropathy*”[Text Word] OR “neuropathic pain”[Text Word])) AND ((“Neoplasms”[Mesh] OR “Antineoplastic Agents”[Mesh]) OR (chemotherapy[Text Word] OR induced[Text Word] OR cipn[Text Word] OR cancer[Text Word]))) AND ((((“Acupuncture”[Mesh] OR “Acupuncture Therapy”[Mesh]) OR “Acupuncture Points”[Mesh]) OR “Acupuncture Analgesia”[Mesh]) OR acupuncture[Text Word]).
Embase: chemotherapy-induced peripheral neuropathy/ OR exp *peripheral neuropathy/OR (peripheral neuropathy* or neuropathic pain).ti,ab,kw. AND exp antineoplastic agent/ae [Adverse Drug Reaction] OR exp neoplasm/ OR (chemotherapy or induced or cipn or cancer).ti,ab,kw. AND acupuncture.ti,ab,kw. or exp acupuncture analgesia/ or exp acupuncture/ or exp acupuncture needle/.
Cochrane Central: [mh “ACUPUNCTURE ANALGESIA”] or [mh ACUPUNCTURE] or [mh “ACUPUNCTURE THERAPY”] or (acupuncture): ti,ab,kw [mh “Peripheral Nervous System Diseases”] or (“peripheral neuropathy*” or “neuropathic pain”): ti,ab,kw [mh “Antineoplastic Agents”] or [mh Neoplasms] or (chemotherapy or cipn or cancer or induced or complication*):ti,ab,k.
Scopus: TITLE (acupuncture) AND TITLE-ABS-KEY (“peripheral neuropathy*” or “neuropathic pain”) AND TITLE-ABS-KEY (chemotherapy or cipn or cancer or induced or complication*).
CINAHL: (MH “Peripheral Nervous System Diseases+”) or “peripheral neuropathy*” or “neuropathic pain” AND (MH “Acupuncture+”) or Acupuncture AND (MH “Antineoplastic Agents+/AE”) or (MH “Neoplasms”) or chemotherapy or induced or cipn or cancer.

The search resulted in a total of 607 articles (Table 1).

**Table 1:** Number of studies found in the databases. Edmonton, AB, Canada, 2018

**Databases**	**Nº of studies (n=xx)**
PubMed*	**146**
**Embase**	**260**
**Cochrane Central**	**38**
**Scopus**	**77**
**CINAHL** ^**†**^	**86**
	**Total: 607**

*PubMed - PubMed of National Library of Medicine; ^†^CINAHL - Cumulative Index to Nursing and Allied Health Literature

**Figure 1:**
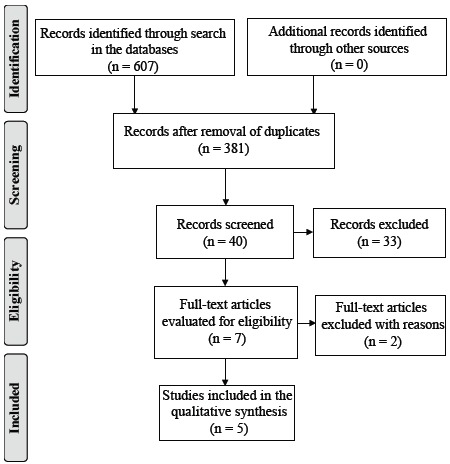
Flowchart, according to PRISMA*, to select the studies found^18^

After the removal of duplicates, all titles and abstracts were reviewed and 7 articles were selected for reading of the full text. Two further articles were removed after the full review because they did not meet inclusion criteria (Figure 1).

*PRISMA - Preferred Reporting Items for Systematic Reviews and Meta-Analyses.

The characteristics of the studies to be analyzed were: title, authors, year of publication, design, evidence level, population and sample size, measure for CIPN, treatment and results, and comments from the authors about specific characteristics of the study. The studies were evaluated according to the level of evidence required for the question of the systematic review. As the question investigated in this review was the effects of a treatment, the following levels of evidence were considered: Level I: systematic reviews; Level II: individual randomized controlled trials; and Level III: quasi-experimental studies, and cohort studies^20^.

The critical evaluation of the studies was done by two independent reviewers, according to the Checklists for Cohort Studies and Quasi-Experimental Studies from the Joanna Briggs Institute Critical Appraisal Tools^21^. These instruments were chosen because they allow the methodological evaluation of the studies and of the scientific evidence found in the proposed systematic review.

## Results

**Figure 2:**
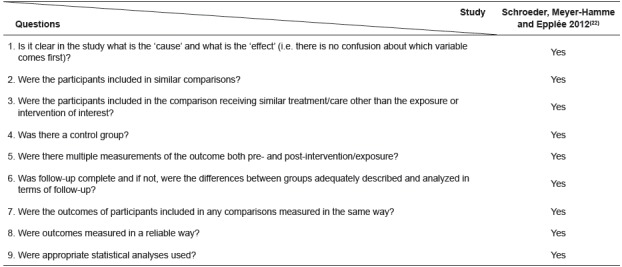
Evaluation of the methodological quality of the quasi-experimental study included in the review according Joanna Briggs Institute Critical Appraisal Tools^21^

**Figure 3:**
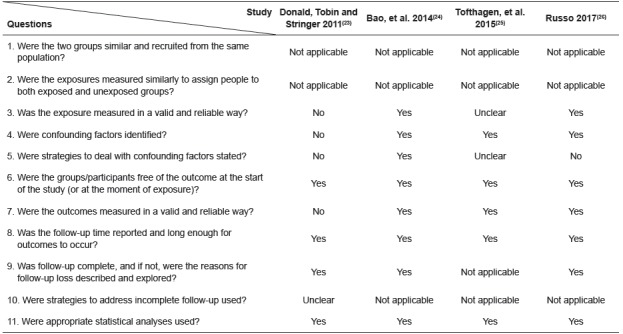
Evaluation of the methodological quality of the cohort studies included in the review according Joanna Briggs Institute Critical Appraisal Tools^21^

The Figures 2 and 3 summarize the characteristics of the quasi-experimental study and of the cohort studies included in the review, according to the assessment of methodological quality from Joanna Briggs Institute Critical Appraisal Tools^21^.

The quasi-experimental study included in this review met all of the criteria outlined by the Joanna Briggs Institute for studies of this design, and thus it is of good quality. One cohort study^23^ did not meet the Joanna Briggs Institute Checklist for cohort studies, indicating significant methodological weaknesses. The weaknesses included use of questionnaires that have not been tested for validity and reliability, and failure to identify and control confounding factors in the analysis. In another cohort study^26^, the authors identified possible confounding factors but did not use strategies to control them. The other cohort studies were well done. 

**Figure 4:**
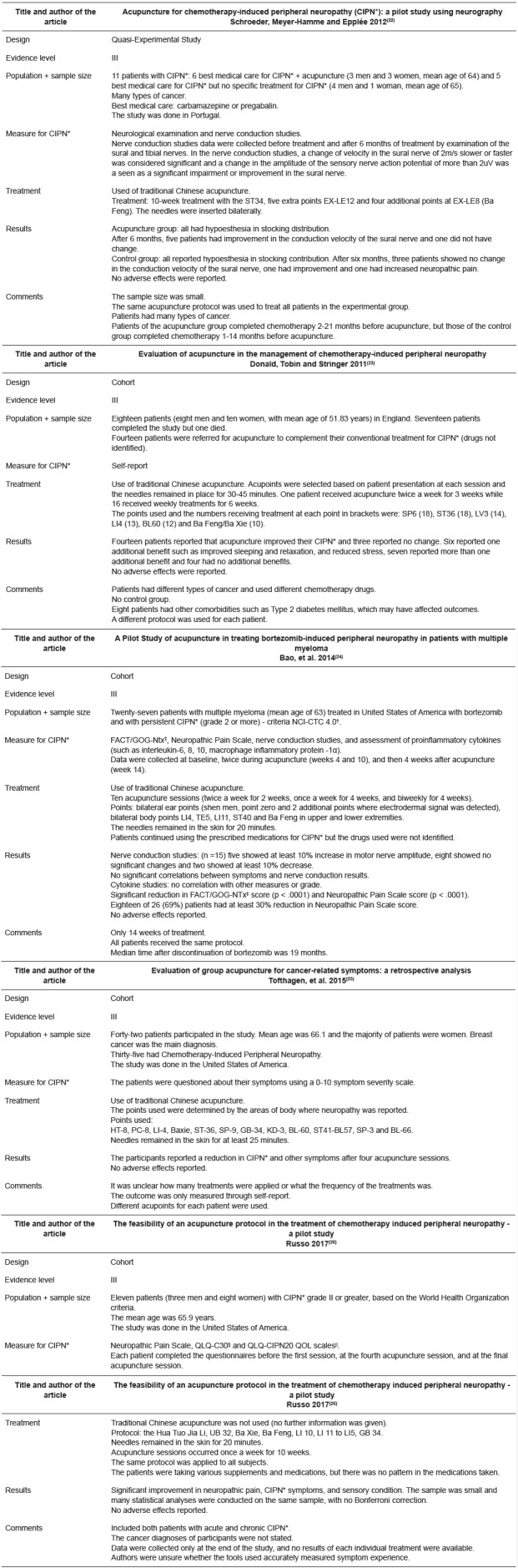
Summary of studies on acupuncture intervention

The articles included in the review are summarized in Figure 4. All authors found that acupuncture had a positive effect on CIPN symptoms in at least some participants, with no adverse events. However, there were also some individuals who did not report a reduction in CIPN symptoms after acupuncture. Based on this review, it seems reasonable to support the use of acupuncture by cancer patients interested in reducing CIPN symptoms.

*CIPN - Chemotherapy-Induced Peripheral Neuropathy; ^†^NCI-CTC 4.0 - National Cancer Institute-Common Toxicity Criteria 4.0; ^‡^FACT/GOG-NTx - Functional Assessment of Cancer Therapy/Gynecologic Oncology Group - Neurotoxicity questionnaire; ^§^QLQ-C30 - Quality of Life Questionnaire; ^||^QLQ-CIPN20 QOL scales - Quality of Life Questionnaire-Chemotherapy-Induced Peripheral Neuropathy 20 Quality of Life scales

## Discussion

One quasi-experimental study and four cohort studies were included in this review. The authors of all studies showed that acupuncture was associated with an improvement in CIPN symptoms in at least some participants and no one experienced adverse events. The cohort and quasi-experimental studies were classified as having evidence level III, which is defined as less reliable studies. The methodological quality of the articles, however, was very good, which indicates that they were good studies of the effects of the intervention in CIPN patients.

The antineoplastic agents used in the studies in this review (Oxaliplatin, Cisplatin, Vincristine and Bortezomib) affect the nervous system differently, and all can reach the ganglion cells of the dorsal roots to the distal axons^27^. The use of different chemotherapeutic agents across the studies included in this review may explain some of the variability in the findings^28^. The authors of one study^28^ also noted that the development and intensity of symptoms were related to the dose of the chemotherapeutic drug, what is something that could have been better explored by the authors of the included studies.

A number of additional factors may have also contributed to the difficulty to compare the findings. First, while some authors^22^ ^,^ ^24^ ^,^ ^26^ used the same acupuncture protocol for all patients, others^23^ ^,^ ^25^ did not do so, but reather adapted the protocol to each patient’s unique problems. Second, in most studies some patients also used medications such as pregabalin and carbamazepine to treat CIPN symptoms during the time they received acupuncture. Medication use was not controlled in the analysis, making it difficult to know whether acupuncture was responsible for the changes in CIPN symptoms or not. Third, the time of the protocols varied considerably from only a few weeks to 14 weeks. Finally, although some acupoints such as Ba Feng, Ba Xie, LI11 and LI4 were used in several studies^23^ ^-^ ^26^, there were no studies using exactly the same acupoints.

The authors of one study^24^ discussed the possible mechanisms of action of acupuncture, but its findings did not support any of these mechanisms. In general terms, analgesia in the context of acupuncture occurs through the activation of a pain control system, which is a complex system involving the stimulation of neurons from different regions of the brain. These neurons send a signal of inhibition of pain to the spinal cord. In this system, there are neurotransmitters such as serotonin, encephalin and endorphin that are also released to aid in the analgesic effect of acupuncture^29^. This may explain why nerve conduction studies had no significant correlation with pain improvement, as these studies merely analyzed the speed and amplitude of action potentials, but did not analyze the substances involved in the process. According to the International Association for the Study of Pain (IASP), neuropathic pain is “the pain caused by injury or disease of the somatosensory system”. It is present in 40% of cancer patients, and CIPN patients have a three-fold greater chance of developing neuropathic pain, a condition that is characteristic of CIPN^30^. This finding supports the importance of identifying some other objective measure of CIPN symptoms. 

The evaluation of CIPN is difficult. In clinical settings, health care providers typically depend on the patient’s subjective report of CIPN symptoms using short questionnaires or other tools^31^. Thus, it is not surprising that most of the authors of the studies included in this review also used subjective measures for their dependent variable.

Two groups of authors in this review^22^ ^,^ ^24^ used both objective measures, such as nerve conduction studies (NCS), and subjective measures to evaluate the effects of acupuncture. Nerve conduction studies measure the amplitude and velocity of conduction of composite motor and sensory action potentials^32^. The use of NCS is problematic because it requires referral to specialized laboratories and causes discomfort to patients^31^. 

In this review, none of the authors were able to demonstrate a correlation between objective and subjective measures of CIPN symptoms. This could be due to low sensitivity of the subjective and objective measures used. Further research on the identification of tools for accurately measuring the symptoms associated with CIPN is urgently needed. Such work should ideally be conducted with scholars fluent in the languages used by those who have studied the effects of acupuncture within the Traditional Chinese Medicine tradition, so that new approaches can be built upon gains made by these individuals.

This study had two main limitations. First, the number of available studies was small, due to limited research in this area. Second, it was difficult to compare results among studies due to differences in outcomes and in measurement tools used. Some research teams used only subjective measures while others used both subjective and objective measures. 

Based on this systematic review, we developed several recommendations for future research. First, the use of an experimental study design is strongly encouraged as it incorporates a control group to which the outcomes in the experimental group can be compared. A related point is the recruitment of a homogenous sample, such as those who are in treatment or those who have finished treatment, large enough to detect possible differences between the experimental and control group. 

Second, it seems from the studies included in this review that there is a dose-response effect between acupuncture and outcome. For this reason it would be interesting to see if patients who receive more sessions over a longer period of time are likely to have better outcomes on both objective and subjective measures.

Finally, better measures for evaluating the impact of acupuncture on CIPN symptoms and quality of life are urgently needed. One group of authors^33^ evaluated eight articles that showed an inverse relationship between CIPN and quality of life, which is not surprising as CIPN is characterized by numbness and burning sensation in lower and upper extremities, which leads to difficulties to drive, write and walk. Researchers planning to conduct studies of acupuncture in the future are, therefore, encouraged to use quality of life instruments to analyze the effect of acupuncture in this important factor in adults with CIPN. 

Subjective measures involving patient report are the gold standard in symptom management. Although objective measures are of interest, the most important symptom measure is the perception of the patient. We recommend the use of robust symptom measures such as the Memorial Symptom Assessment Scale (MSAS), with the assessment of severity and frequency of the symptoms, and distress. In addition, quality of life should always be included as a dependent variable, as CIPN appears to significantly reduce the ability to perform activities of daily life. 

## Conclusion

This systematic review was based on five studies about the use of acupuncture as intervention for improving the symptoms of chemotherapy-induced peripheral neuropathy. Acupuncture appears to be an effective intervention for treating some adult CIPN patients and is not associated with adverse events. More experimental studies with larger and more homogeneous samples over longer periods of time are urgently needed. In addition, it is important to develop new measurement approaches for the assessment of CIPN symptoms and include quality of life as an outcome measure. 
